# Phenolic, Headspace and Sensory Profile, and Antioxidant Capacity of Fruit Juice Enriched with *Salvia officinalis* L. and *Thymus serpyllum* L. Extract: A Potential for a Novel Herbal-Based Functional Beverages

**DOI:** 10.3390/molecules28093656

**Published:** 2023-04-22

**Authors:** Ivanka Maleš, Ana Dobrinčić, Zoran Zorić, Sanda Vladimir-Knežević, Ivona Elez Garofulić, Maja Repajić, Danijela Skroza, Igor Jerković, Verica Dragović-Uzelac

**Affiliations:** 1 Department of Pharmacy, School of Medicine, University of Split, Šoltanska 2, 21000 Split, Croatia; ivanka.males@mefst.hr; 2 Faculty of Food Technology and Biotechnology, University of Zagreb, Pierottijeva 6, 10000 Zagreb, Croatia; zoran.zoric@pbf.unizg.hr (Z.Z.); ivona.elez@pbf.unizg.hr (I.E.G.); maja.repajic@pbf.unizg.hr (M.R.); vdragov@pbf.hr (V.D.-U.); 3 Department of Pharmacognosy, Faculty of Pharmacy and Biochemistry, University of Zagreb, Marulićev trg 20, 10000 Zagreb, Croatia; svladimir@pharma.hr; 4 Department of Food Technology and Biotechnology, Faculty of Chemistry and Technology, University of Split, Ruđera Boškovića 35, 21000 Split, Croatia; danci@ktf-split.hr; 5 Department of Organic Chemistry, Faculty of Chemistry and Technology, University of Split, Ruđera Boškovića 35, 21000 Split, Croatia; igor@ktf-split.hr

**Keywords:** sage, wild thyme, functional beverages, fruit juices, bioactive compounds, phenolics, headspace composition, antioxidant capacity, sensory properties

## Abstract

Since certain constituents are not naturally present in pure fruit juices, incorporating herbal extracts can provide specific sensory properties to the beverages and improve their biopotential. In our previous research, it was found that sage (*Salvia officinalis* L.), wild thyme (*Thymus serpyllum* L.), and combinations of their extracts had the highest total phenolic content and a unique composition of volatile compounds, which can contribute to the aromatic and antioxidant qualities of functional products. Therefore, this research aimed to investigate the potential of sage and wild thyme extracts, as well as their mixture (wild thyme:sage at 3:1, *v*/*v*), to enrich fruit juices (apple, pineapple, and orange). Obtained beverages were evaluated for sensory properties as well as phenolic and headspace composition (UPLC-MS/MS and HS-SPME/GC-MS analysis) and antioxidant capacity (ORAC assay). The incorporation of wild thyme extract in pineapple juice provided the most harmonious flavor and the highest content of volatile compounds (on PDMS/DVB fiber). The orange juice formulations were the most enriched with phenolic and volatile compounds (on DVB/CAR/PDMS fibers). The highest antioxidant capacity was observed in the formulation with orange juice and sage extract (22,925.39 ± 358.43 µM TE). This study demonstrated that enriching fruit juices with sage and wild thyme extracts could create functional beverages with improved sensory and health-promoting properties, providing valuable insights for the food and beverage industry to meet the growing demand of health-conscious consumers for natural and functional products.

## 1. Introduction

Functional beverages are one of the most remarkable and rapidly expanding segments in the functional food sector. The development of functional beverages faces numerous challenges, such as creating new formulations or improving the existing products to achieve greater health benefits or better sensory properties [1,2,3,4]. Market and consumer trends have shown that there is an increasing demand for low-calorie carbonated beverages and functional beverages composed of herbal extracts, which are a large source of bioactive molecules responsible for a variety of beneficial properties and health-promoting effects [3,5,6,7]. Phenolic compounds are among the most abundant and widely distributed classes of secondary plant metabolites and are therefore an important component of the human diet [8]. In recent decades, the interest in food phenolic compounds has greatly increased due to their antioxidant power. Natural phenolic compounds have gained popularity over artificial antioxidants, mainly because consumers prefer functional foods and beverages with natural ingredients that retain their flavor and nutritional value [9,10]. The ability of phenolic compounds to scavenge free radicals contributes to the prevention of oxidative stress-related chronic disorders, such as cancer, cardiovascular, and neurological diseases [11]. The use of medicinal and aromatic plants for the production of functional beverages is very challenging, as the desired final product must be rich in bioactive molecules and have acceptable sensory properties. When selecting herbal extracts for use in functional beverages, the composition of bioactive molecules, their synergistic effects, and the type of beverage to be prepared must be considered [3,7]. Usually, herbal extracts are incorporated into beverages in small amounts at the pre-mix or syrup stage, along with other ingredients such as colorants, flavorings, high-intensity sweeteners, and preservatives [12]. The combination of medicinal and aromatic herbal extracts with beverages containing significant amounts of sugar, salt, and/or citric acid may mask the aroma of the beneficial addition due to the bitter reputation of these extracts [13]. In recent years, fruit juices have become one of the most notable areas of the functional beverage market [14,15]. Juice blends are an interesting approach to adding new flavors to the beverage industry, which is demanding novel products [16]. Fruit juices can be enriched with herbal extracts to increase the biological value of the beverage but also to improve its aroma, flavor, and nutritional value [17]. It is believed that the addition of herbal extracts to fruit juice has no negative impact on consumer acceptance of the juice [18,19]. Moreover, numerous studies have demonstrated that the addition of herbal extracts to fruit juices has a favorable effect on their sensory properties [20,21,22]. Yellow, orange, and red fruit juices are usually enriched with herbal extracts to enhance their naturalness without altering the flavor or color [12]. Apple (*Malus domestica* Borkh.), pineapple (*Ananas comosus* (L.) Merr.), and orange (*Citrus sinensis* L.) are the most popular fruits consumed in the functional beverage industry due to their bioactives, delicious flavor, and aroma. Therefore, their juices are expected to be able to attenuate the distinctive aroma of the herbal extract [23,24,25]. Consumption of fruit-based beverages rich in phenolic compounds is also associated with healthy diets such as the Mediterranean diet and the prevention of chronic diseases because of their antioxidant properties [26]. In addition, phenolic compounds play an important role in determining the color and flavor of a product; hence, they can be easily oxidized [27]. The addition of herbal extracts to fruit juices can also contribute to a completely new flavor of the beverage, so it is of great importance to evaluate the sensory acceptability of the beverage [28]. The aroma characteristics present in the herbal extracts depend on the volatile compounds, which also have a significant impact on the sensory properties. They are especially important for customer acceptance of the product and are of particular significance in the development of functional beverages, as their degradation during thermal treatment can lead to loss of flavor and odor [29,30].

Mediterranean herbs from the Lamiaceae family, including sage (*Salvia officinalis* L.) and wild thyme (*Thymus serpyllum* L.), have a remarkable composition of bioactive molecules such as hydroxycinnamic acids, flavones, and terpenes, which may influence the antioxidant properties and sensory qualities of the beverage [31,32,33]. The extracts of these herbs also possess a wide range of biological properties that are advantageous for their biomedical applications, including antibacterial, antifungal, anti-inflammatory, antidiabetic, and anticancerogenic activities [34,35,36,37]. Their beneficial biological characteristics are valuable for any prospective biomedical uses; however, their application as a substitute for currently used medicines requires extensive additional research.

Sage, wild thyme, and their two-component extract mixture were found to have the highest total phenolic content of all samples in our previous study of laurel (*Laurus nobilis* L.), sage, and wild thyme extracts. They also have a unique composition of volatile compounds that may contribute to the aromatic qualities of the final products prepared from these herbal extracts [38]. According to the literature, there has been a lack of studies in the past decade comparing formulations with apple, pineapple, or orange juice fortified with sage, wild thyme, or wild thyme with sage extract. The aim of this study was therefore to analyze for the first time the potential of using individual sage and wild thyme extracts as well as a wild thyme and sage (3:1, *v*/*v*) mixture to fortify apple, pineapple, and orange juices, to evaluate their sensory properties, and to characterize their phenolic and headspace composition as well as antioxidant capacity.

## 2. Results and Discussion

### 2.1. Sensory Evaluation

Sensory analysis is frequently used in food science and technology to evaluate the product’s quality in terms of intrinsic quality indicators [39]. Fruit juice can be balanced in terms of its color, acidity, flavor, and palatability by blending it with herbal extract to produce consumer-pleasing products [40]. In addition, the enrichment of fruit juice with herbal extracts provides an opportunity to develop new attractive products, which is of particular interest to those consumers who follow new trends in the functional beverage industry [41]. According to previous findings, apple, pineapple, and orange juices were enriched with different herbal extracts [20,42,43,44,45]. Ivanišová et al. [46] reported that the enrichment of apple juice with sage and wild thyme extract offered better sensory properties than 100% apple juice. However, to the authors’ knowledge, there has been no study comparing formulations with apple, pineapple, or orange juice enriched with sage, wild thyme, and/or wild thyme with sage extract. Therefore, this study provides new insights into the potential use of these Mediterranean plant species in the production of functional beverages. The combination of fruit juices and herbal extracts is important in terms of their biological properties, but sensory features are one of the critical attributes that determine whether the final product will be accepted by consumers. In the sensory evaluation of food products, color is the most important criterion for assessing juice quality and a key indicator of potential consumer preference [47]. In addition, color, odor, flavor, and aroma of fruit juice are among the most important sensory quality parameters and are of great importance for the consumer’s acceptance of the juice [42]. Many factors influence the sensory characteristics of fruit juice, including individual biological factors (e.g., saliva characteristics and genetics), climate and agricultural practices for fruit growing, technological factors for juice preparation (e.g., juice processing methods, storage), and the content of various compounds, especially carbohydrates [48,49]. In this study, sensory analysis was performed on functional beverages prepared from apple juice (*AJ*), pineapple juice (*PJ*), and orange juice (*OJ*), to which sage (*S*), wild thyme (*WT*), and wild thyme:sage (3:1, *v*/*v*) (*WTS*) extracts were added at 5%, 10%, and 15%, respectively. The influence of juice type, extract, and extract concentration on color intensity, odor, flavor, and aroma was evaluated, and the results are shown in Table 1. 

*PJ* was found to have the most intense color (7.72), and color intensity increased with increasing extract concentration. In addition, *PJ* had the most intense odor among the juices (6.21), and formulations containing *PJ* had the most harmonious flavor (8.70). The most intense odor, flavor, and aroma of *S* as well as the odor of *WT* were found in beverages with *AJ* (2.08, 2.38, 2.51, and 2.57), which were also the sweetest (6.56). The beverages prepared with *OJ* were more sour (3.29), bitter (1.98), and the *WT* aroma was more profound (2.83). With the exception of the flavor of *WT*, which was stronger in beverages containing *WTS* mixture (4.10), the odor, flavor, and aroma of *S* or *WT* were generally higher in the corresponding beverages (2.97, 4.19, 4.44 for *S*, and 3.47 and 4.08 for *WT*). Juice aroma and flavor were highest in the beverages with the highest *WT* (6.48 and 6.71, respectively). The beverage formulations containing *WT* and *S* were rated as more sour and more bitter, respectively (2.43 and 1.44). As expected, when the extract concentration was higher, the odor, aroma, and flavor were more intense. In general, the extract odor, flavor, and aroma received lower average scores (were less profound) when compared with the attributes deriving from juice because they were used only at concentrations of 5%, 10%, or 15%.

In the present study, a new type of enriched fruit juice is presented, and it was found that *WT* extract, *S* extract, and their mixture can be well incorporated into fruit juices and accepted by consumers. Based on the average scores of all evaluators, the samples with 10% extract showed the best results, so these samples were used for additional ultra-performance liquid chromatography-tandem mass spectrometry, headspace solid-phase microextraction, and antioxidant analyses.

### 2.2. Phenolic Characterization

The phenolic profile of *AJ*, *PJ*, *OJ*, apple juice + sage extract (*AS)*, apple juice + wild thyme extract *(AWT*), apple juice + wild thyme:sage (3:1, *v*/*v*) extract (*AWTS*), pineapple juice + sage extract (*PS*), pineapple juice + wild thyme extract (*PWT*), pineapple juice + wild thyme:sage (3:1, *v*/*v*) extract (*PWTS*), orange juice + sage extract (*OS*), orange juice + wild thyme extract (*OWT*), and orange juice + wild thyme:sage (3:1, *v*/*v*) extract (*OWTS*) was analyzed by ultra-performance liquid chromatography-tandem mass spectrometry (UPLC/MS-MS). The results (Table 2) showed the presence of 29 phenolic compounds, including flavonoids (flavones, flavonols, flavan-3-ols, and proanthocyanidins), hydroxycinnamic, and hydroxybenzoic acids.

Among the hydroxycinnamic acids, compounds **10**, **11**, **15**, **20**, and **26** (Table 2) were quantified as chlorogenic acid, caffeic acid, *p*-coumaric acid, ferulic acid and rosmarinic acid, respectively, by comparison with authentic standards. Only *AJ* demonstrated the presence of chlorogenic acid in a noticeably higher concentration. The most abundant acid in all formulations containing *S* was caffeic acid. Previous studies reported the presence of caffeic acid in pineapple extract [50,51], orange peel extract [52], apple extract [53], and sage extract [36,38,54,55,56], as well as in pineapple juice enriched with pine (*Pinus pinaster* Ait.) bark extract [57]. The formulations with the highest concentrations of rosmarinic acid were *PWT*, *PWTS*, *OWT,* and *OWTS*, whereas those with the highest concentrations of chlorogenic acid were *AWT* and *AWTS*. Since rosmarinic acid is not a characteristic constituent of *PJ* and *OJ*, its presence in the formulations is attributed to *WT* and *WTS* extracts, which have a significant content of rosmarinic acid according to a previous study [38]. However, the presence of rosmarinic acid was previously reported in pineapple mixed beverages [58]. Chlorogenic acid has been described as one of the leading phenolic compounds in apple juice [59,60,61], and its presence was high in all apple formulations, particularly in *AWT*. Moreover, it constitutes the majority of phenolic acids in all samples.

As for the hydroxybenzoic acids, compounds **7, 9,** and **29** were quantified through comparison with authentic standards such as protocatechuic, syringic, and gallic acid. Based on the fragment ion at 155 *m*/*z* and the fragmentation loss of −162 amu, which is characteristic of a hexose residue [62], the compound **6** was tentatively quantified as 3,4-dihidrobenzoic acid hexoside. Compound **8** was assigned as *p*-hydroxybenzoic acid based on the previously described fragmentation pattern [63]. Syringic acid predominated in all fruit juices, primarily in *PJ*, and it was even higher in every formulation, with the exception of *AJ,* which had slightly higher concentrations of gallic acid. The presence of syringic acid has already been reported for pineapple extract [50], *Thymus* species [38,64,65], and sage extract [34,66].

Among the flavones, compounds **18** and **28** were quantified by comparison with authentic standards such as luteolin and apigenin. Based on the precursor ion at *m*/*z* 449 and the fragment ion at *m*/*z* 329, which correspond to the loss of −120 amu, characteristic of the hexose residue in C-glycosylation [67], compound **12** was tentatively quantified as luteolin-6-C-hexoside. The precursor ion at *m*/*z* 579 and the fragment ion at *m*/*z* 459, both comparable to the fragmentation pattern previously described by Pacifico et al. [68], led to a preliminary quantification of compound **1** as apigenin-6-C-(*O*-deoxyhexosyl)-hexoside. Luteolin was the dominant compound in all fruit juices, especially in *OJ* and its formulations. The presence of luteolin in orange peel extract has already been reported [52], as well as in sage and wild thyme extracts [37,38,69].

By comparison with the authentic standards, compounds **13, 14,** and **21** from the flavonol class were quantified as myricetin, quercetin-3-glucoside, and rutin, respectively. The particular fragment ion at *m*/*z* 287 that is characteristic for kaempferol was used to quantify the compounds **4**, **16**, **17,** and **22**. Because of the specific fragment loss, they were tentatively quantified as the following compounds: kaempferol-3-*O*-deoxyhexoside (deoxyhexose −146 amu), kaempferol-3-rutinoside (rhamnose −146 amu; hexose −162 amu), kaempferol-3-*O*-hexoside (hexose −162 amu), and kaempferol-3-*O*-pentoside (pentose −132 amu) [70]. Compounds **23** and **25** were tentatively assigned according to the characteristic fragment ion at *m*/*z* 303 and specific loss of sugar moieties as quercetin-3-pentoside (pentose −132 amu) and quercetin-3-rhamnoside (rhamnose −146 amu). Compound **19** was quantified as isorhamnetin-3-hexoside by the precursor ion at *m*/*z* 479 and the fragment ion at *m*/*z* 317, corresponding to the loss of hexose (−162 amu). Flavonols were found to be the most dominant compounds among all polyphenols, especially rutin, which was found in impressive amounts in *OJ* and all orange formulations. Rutin exhibits numerous pharmacological activities and has been described as one of the major flavonoids in orange peel and as an essential nutritional constituent of plant-based foods [71,72]. The concentration of rutin was highest in the *OWTS* formulation, which is consistent with our previous reports showing the highest rutin content in the *WTS* mixture [38]. All formulations contained significant amounts of other flavonols such as quercetin and kaempferol derivatives.

In terms of flavan-3-ols, compounds **3**, **24,** and **27** were found to be catechin, epicatechin gallate, and epigallocatechin gallate, respectively, by comparison with authentic standards. Based on the precursor ion at *m*/*z* 291 and the fragment ion at *m*/*z* 123, compound **5** was tentatively quantified as epicatechin. Catechin and epicatechin were the major flavan-3-ols in the samples, although their concentrations were lower than those of other polyphenols. They have previously been found in pineapple peel [73], orange peel extract, and apple fruit [74,75], as well as in sage and wild thyme extracts [38,76,77].

Among proanthocyanidins, only compound **2** was found, which was tentatively quantified as a procyanidin trimer based on the precursor ion at *m*/*z* 865 and the fragment ion at *m*/*z* 575 formed by the heterocyclic ring system subunits’ retro Diels-Alder (RDA) fission, described earlier [78]. Proanthocyanidins were found mainly in *PJ*, as previously confirmed by Luximon-Ramma et al. [79].

### 2.3. Headspace Solid-Phase Microextraction (HS-SPME/GC-MS)

In *AJ*, *PJ*, *OJ*, *AS*, *AWT*, *AWTS*, *PS*, *PWT*, *PWTS*, *OS*, *OWT,* and *OWTS*, volatile headspace compounds were isolated and analyzed by HS-SPME/GC-MS, and the results are shown in Table 3 and Table 4.

A total of fifty-nine compounds were identified using PDMS/DVB fiber (nine aliphatic alcohols, three aldehydes, one alkane, two esters, three ketones, thirty-four monoterpenes, four benzene derivatives, and three sesquiterpenes), and a total of forty-nine compounds were identified using DVB/CAR/PDMS fiber (eight aliphatic alcohols, four aldehydes, three ketones, twenty-seven monoterpenes, four benzene derivatives, and three sesquiterpenes). Using PDMS/DVB fiber, seven volatile compounds were identified in *AJ*, five in *PJ,* and eighteen in *OJ*. Ethanol (61.73%) was the most abundant volatile compound in *AJ*, followed by propan-2-one (61.82%) in *PJ,* and limonene (92.24%) in *OJ*. Previous studies have reported high ethanol concentrations in apple juice, possibly due to the exposure of apple fruit to anaerobic or hypoxic conditions [80,81,82]. The presence of propan-2-one has also been reported in pineapple juice, which is likely due to thermal degradation [83]. In addition, limonene contributes to orange fragrance and has been previously described as the most abundant volatile compound in orange peel and one of the major constituents in processed orange juice [84,85]. The highest number of volatile compounds was found in the *PWT* formulation (40). Among *S* extracts, the *OS* formulation had the highest number of volatile compounds (10), while in the *WTS* mixture, the highest number of volatile compounds was present in *AWTS* formulation (30).

(*Z*)-β-Ocymene (12.18%) was the main compound found in the *PWT* formulation. Its presence was previously reported in the essential oil of *Thymus algeriensis* [86]. Other compounds detected in high concentrations in the *PWT* formulation were carvacrol (9.09%), followed by thymoquinone (8.94%), and 4-terpineol (8.74%). Moreover, 4-terpineol was the main compound found in the *AWT* formulation (15.28%). Its presence has been demonstrated in wild thyme extract and essential oil [38,87]. Carvacrol and borneol were the compounds found in high concentrations in the *AWT* formulation (11.74 and 10.19%, respectively). In previous studies, carvacrol has been described as the major constituent of wild thyme extract and essential oil [38,88,89] and as the major constituent of hydrolates of other *Thymus* species such as *Thymus vulgaris* and *Thymus capitatus* [90,91]. The presence of borneol was previously reported in wild thyme extract, essential oil, and hydrolate [38,92,93]. Monoterpenes, especially β-thujone, were the predominant compounds in *AS* (34.72%), *PS* (30.63%), *AWTS* (21.93%), and *PWTS* formulations (17.74%). β-Thujone was previously described as the dominant compound in sage extract and essential oil [38,94,95]. Other compounds detected in high content in these formulations were α-thujone, 1,8-cineole, and camphor. All of the herbal extracts mixed with *OJ* did not contain any compounds that had a significant effect on the aroma, with the exception of limonene; therefore, limonene is the main contributor to the pleasant aroma [96] of these formulations. In addition, limonene has been previously described as the compound with an appreciable antioxidant activity [97,98]. The previously mentioned compounds also have a great influence on the sensory properties of the product. For example, 4-terpineol provides fruity and floral notes [81], and carvacrol provides a thymol-like, warm and spicy flavor [99]. In addition, borneol provides a spicy, fragrant, cool flavor and herbaceous odor [100,101], and β-thujone provides a camphor-like, herbal odor [102]. In addition, these compounds have also shown a broad spectrum of health benefits besides antioxidant activity, such as antimicrobial, anticancer, antifungal, anti-inflammatory, cardioprotective, and neuroprotective effects [81,103,104,105,106,107,108,109,110,111,112].

Using DVB/CAR/PDMS fiber, seven compounds were identified in *AJ*, with ethanol (47.95%) as the dominant compound. Only three compounds were identified in *PJ*. Interestingly, 3-methylbutanal, an intermediate of the Ehrlich pathway [113], was the predominant compound on this fiber (80.11%), whereas it was not detected when using PDMS/DVB fiber. In *OJ,* 16 compounds were identified, with limonene being the dominant compound (91.12%). The highest number of detected compounds from all herbal extracts was present in the formulation with *OJ*. A total of 27 compounds were found in *OWT*, 22 in *OS,* and 26 in the *OWTS* formulation. Carvacrol dominated in *AWT* (17.83%) and *PWT* formulations (18.97%). In the other formulations [*AS* (35.68%), *PS* (31.65%), *AWTS* (20.11%), and *PWTS* (10.83%)], β-thujone dominated, except for the formulations with *OJ*, in which limonene was the dominant compound. Other compounds detected in high concentrations in these formulations were α-thujone, camphor, 4-terpineol, and ethanol.

Almost every compound detected in this study was present at lower concentrations in formulations with a two-component herbal extract mixture. However, there are some exceptions, as some of the compounds are characteristic only of single herbal species, so their concentration was expected to increase when two herbal extracts were mixed. On PDMS/DVB fiber, 1,8-cineole and β-thujone were present in higher concentrations in *AWTS* (9.03%; 21.93%) than in the *AWT* formulation (1.48%; 1.91%). The presence of linalool and borneol was higher in *AWTS* (3.71%; 5.97%) than in the *AS* formulation (1.06%; 3.28%). The percentage of camphor was higher in *AWTS* and *PWTS* (13.10%; 12.97%) than in *AWT* and *PWT* formulations (5.72%; 2.83%). 4-Terpineol was detected in higher concentrations in *AWTS* and *PWTS* (7.46%; 7.14%) than in *AS* and *PS* formulations (1.92%; 1.74%).

On DVB/CAR/PDMS, fiber linalool was present at a higher percentage in *AWTS* and *PWTS* (4.40%; 3.06%), than in *AS* and *PS* formulations (1.13%; 1.12%). Camphor was detected in greater amounts in *PWTS* (10.05%) than in the *PWT* formulation (2.06%). Borneol was present in a higher percentage in *AWTS* (5.96%) than in the *AS* formulation (3.05%). The presence of 4-terpineol was higher in *AWTS* and *PWTS* (9.78%; 7.94%) than in *AS* and *PS* formulations (2.67%; 2.55%). Limonene was detected in a higher percentage in *OWTS* (82.30%) than in *OS* (72.50%) and *OWT* (81.43%) formulations. However, the amount of borneol in *PWTS* (5.94%; 5.57%) was higher than in *PS* (3.02%; 3.54%) and *PWT* (5.30%; 5.32%) formulations on PDMS/DVB and DVB/CAR/PDMS fibers, respectively.

In addition, a total of 25 compounds were identified on PDMS/DVB fiber, and a total of 19 compounds were identified on DVB/CAR/PDMS fiber at levels less than 1%, mainly monoterpenes and alcohols.

To our knowledge, the headspace analysis of blended apple, pineapple, and orange juice with sage and wild thyme extracts and their mixture has not yet been performed. However, Souza et al. [114] enriched apple juice with cardamom tea (*Elettaria cardamomum* L. Maton) and identified a total of 46 volatile compounds, among which esters and ethers were predominant. In addition, Saad et al. [115] identified a wide range of volatiles (hydrocarbons, aldehydes, esters, ketones, fatty acids, phenol derivatives, sulfur compounds) when supplementing cucumber (*Cucumis satibum* L.) juice with cinnamon (*Cinnamomum verum* L.), clove [*Syzygium aromaticum* (L.) Merr. & L.M. Perry], mint (*Mentha spicata* L.), and ginger (*Zingiber officinale* L.) herbal extracts. Kostyra et al. [116] detected 19 compounds, mainly alcohols, aldehydes, esters, and ketones, in mixtures of apple and pear (*Pyrus communis* L.) juice with kiwiberry (*Actinidia arguta* Planch.) juice. Elwakeel and Hussein [117] identified a total of 30 volatile compounds, mostly alcohols, in a mixture of juice from two cactus pear species (*Opuntia ficus-indica* L. and *Opuntia lindheimeri* Engelm.) and guava (*Psidium guajava* L.) juice. There is a possibility of interactions between the volatile compounds, which may have additive or competitive effects on the aroma profile through synergistic or antagonistic actions [118]. The physicochemical properties of the compounds and the bonds that may occur between them have a great influence on these interactions [119]. Moreover, previous studies have shown that volatiles present in lower amounts can nevertheless have a major impact on the product’s aroma due to their synergistic properties [120], making their presence important for overall sensory evaluation.

### 2.4. Antioxidant Capacity

One of the most widely used techniques for evaluating the antioxidant activity of food products is the oxygen radical absorbance capacity (ORAC) assay. This method is based on a hydrogen atom transfer reaction mechanism and uses biologically relevant free radicals, so it can be used to evaluate free radical scavenging activity in various biological systems [121,122]. The results of the ORAC assay in fruit juices and their formulations with herbal extracts of *S*, *WT,* and *WTS* are shown in Table 5.

In our previous study, we demonstrated high antioxidant capacity in herbal extracts of *S*, *WT,* and *WTS* using the ORAC assay, which was due to their high content of bioactive compounds [38]. In the present study, the formulations containing *S*, particularly *OS* formulations, had the highest antioxidant capacity (22,925.39 ± 358.43 µM TE). This is most likely due to the previously described high content of the volatile compound limonene, as well as non-volatile hydroxycinnamic acids and flavonoids such as rutin, kaempferol-3-*O*-hexoside, and kaempferol-3-rutinoside, all of which have been shown to be potent free radical scavengers [123,124]. The ability of polyphenols to scavenge free radicals is related to the substitution of hydroxyl groups in their aromatic rings [125]. In addition, phenolic compounds are assumed to be the major contributors to antioxidant activity in fruit juices [126,127]. Moreover, the synergistic interactions between rutin hydrate and kaempferol in terms of antioxidant activity have been previously reported [128]. In addition, the interactions between hydroxycinnamic acids were observed. Thus, previous research revealed synergistic effects between rosmarinic and caffeic acids as well as between rosmarinic acid and quercetin [129]. Rutin and caffeic acid have been reported to have rather less potent interactions [130]. Total phenolic content and total antioxidant activity in phytochemical extracts from various fruits may be directly correlated. The fruits with higher total phenolic contents have been found to possess enhanced antioxidant effects [131]. Moreover, other studies have confirmed our findings by showing that the addition of herbal extracts to fruit juices increases antioxidant activity as determined by the ORAC assay [132,133]. In addition to phenolic compounds, other substances that can interact with short-lived peroxide radicals, such as sugars or ascorbic acid, also contribute significantly to the antioxidant activity measured by the ORAC assay [133,134]. However, with the exception of the *AWTS* formulation (7397.93 ± 85.56µM TE), where the results were higher than *AWT* (7818.99 ± 44.93 µM TE) but lower than *AS* (9981.77 ± 644.10 µM TE), the formulations with the two-component *WTS* extract did not result in increased antioxidant activity. The dilution effect that may occur when herbal preparations are combined results in a reduction in bioactive components and lower antioxidant activity.

## 3. Materials and Methods

### 3.1. Chemicals

Ethanol, sodium phosphate dihydrate, disodium dihydrogen phosphate, 6-hydroxy-2,5,7,8-tetramethylchroman-2-carboxylic acid (Trolox), fluorescein 3’,6’-dihydroxyspiro[isobenzofuran-1(3H),9’-[9H]xanthen]-3-one and 2,20-azobis(2-methylpropionamidine) dihydrochloride (AAPH) were obtained from Sigma-Aldrich (Steinheim, Germany). Authentic standards of myricetin, caffeic acid, gallic acid, ferulic acid, protocatechuic acid, syringic acid, rosmarinic acid, chlorogenic acid, *p*-coumaric acid, and quercetin-3-glucoside were purchased from Sigma-Aldrich (St. Louis, MO, USA). Catechin, epigallocatechin gallate, epicatechin gallate, kaempferol-3-glucoside, rutin, apigenin, procyanidin B2, and luteolin were obtained from Extrasynthese (Genay, France).

### 3.2. Herbal and Juice Material

The samples of *S* and *WT* were purchased from Suban Ltd. (Strmec Samoborski, Croatia), a certified collector and producer of medicinal and aromatic plants. The plants were harvested in 2020, stored in their original packages (paper bags), and kept in a dry and dark place. Before the extraction, the herbs were ground using an electric grinder (WSG30, Waring Commercial, Torrington, CT, USA). The concentrated apple (*AJ*), pineapple (*PJ*), and orange (*OJ*) juices were obtained from juice producer Stanić Beverages Ltd. (Zagreb, Croatia).

### 3.3. Herbal Extract Preparation

The aqueous herbal extracts *S* and *WT* were prepared according to the optimal conditions established in our previous study [38], where we explored different ratios of two- and three-component herbal extract mixtures containing sage, wild thyme, and/or laurel. A two-component wild thyme:sage (3:1, *v*/*v*) extract mixture showed the highest total phenolic content, as well as a one-component sage and wild thyme extract; therefore, these extracts were chosen for additional research. All samples were prepared in duplicate and stored at 4 °C (for no longer than 7 days).

### 3.4. Functional Beverages Preparation

Concentrated *AJ*, *PJ,* and *OJ* were diluted with water to approximately 11% soluble dry matter and enriched with *S*, *WT,* and *WTS* extracts. Each extract was added to each juice at 5%, 10%, and 15%. Soluble dry matter was measured using Pocket Refractometer PAL-3 (Atago, Japan). Table 6 shows various beverage formulations from fruit juices enriched with herbal extracts.

### 3.5. Sensory Evaluation

The sensory evaluation of enriched fruit juices was carried out by a panel group consisting of 10 trained examiners (5 females and 5 males) in a sensory laboratory set up according to the standards ISO 8589 (2007). In order to become familiar with the product and identify the descriptors to be used, panelists were trained in a two-hour session before the sensory evaluation. A scale from 1 to 10 (1—low intensity, 10—strong intensity) was used to quantify the intensity of the sensory attributes. Each evaluator noted the intensity of each sensory property with a score from 1 to 10 for each of the samples. The following sensory characteristics of the juices enriched with herbal extracts were evaluated: intensity of color, *S*, *WT*, and juice (apple, pineapple, or orange) odor, flavor, and aroma; sweet, sour, and bitter flavor; flavor harmony; and off-flavor. Sensory evaluation was performed on freshly prepared enriched fruit juices. All samples were labeled, pre-tempered to room temperature, and filled into transparent plastic jars with a volume of 100 mL immediately before evaluation. Each sample was subjected to sensory analysis in two sessions. During each session, tap water was provided to examiners to cleanse the mouth and neutralize any flavors.

### 3.6. UPLC-MS/MS Chromatography

An ultra-performance liquid chromatography (UPLC) system (Agilent series 1290 RRLC equipment, Agilent, Santa Clara, CA, USA) was used to separate the targeted phenolic compounds using a Fortis C18 column measuring 100 × 2.1 mm with 1.7 m particle size (Fortis Technologies Ltd., Neston, UK). Elez-Garofulić et al. [135] have previously provided descriptions of gradient settings and eluent compositions. A 64,300 QqQ mass spectrometer (Agilent) was used for the identification and quantification of the phenolic compounds in both ionization modes. In summary, ESI ion source ionized the analytes at a flow rate of 11 L h^−1^ and a temperature of 300 °C while using N_2_ as desolvation and collision gas. Capillary voltage was set at +4 and −3.5 kV and nebulizer pressure at 40 psi. Instrument control and data analysis were performed using Agilent MassHunter Workstation Software (v. B.04.01, Agilent, Santa Clara, CA, USA). The calibration curves of the standards served as the basis for identification and quantitative determination: myricetin, rutin, caffeic acid, gallic acid, ferulic acid, protocatechuic acid, syringic acid, rosmarinic acid, chlorogenic acid, *p*-coumaric acid, quercetin-3-glucoside, kaempferol-3-glucoside, catechin, epigallocatechin gallate, epicatechin gallate, apigenin, procyanidin B2, and luteolin. The identification of the compounds for which there are no reference standards was based on their mass spectral data and mass fragmentation pattern reports published in the literature, while quantification was performed as follows: kaempferol-3-rutinoside, kaempferol-3-*O*-hexoside, kaempferol-3-*O*-deoxyhexoside and kaempferol-3-*O*-pentoside were calculated corresponding to kaempferol-3-glucoside, apigenin-6-C-(*O*-deoxyhexosyl)-hexoside corresponding to apigenin, luteolin-6-C-hexoside according to luteolin, isorhamnetin-3-hexoside, quercetin-3-rhamnoside and quercetin-3-pentoside corresponding to quercetin-3-glucoside, epicatechin corresponding to catechin, 3,4-dihydroxybenzoic acid hexoside according to protocatehuic acid, while *p*-hydroxybenzoic acid was calculated as gallic acid equivalent. All analyses were performed in duplicate, and the concentrations of analyzed compounds are expressed as mean values ± standard error of mg L^−1^ of the sample (*n* = 2 replicates).

### 3.7. HS-SPME/GC-MS

HS-SPME was performed using a manual SPME holder and PDMS/DVB and DVB/CAR/PDMS fibers purchased from Supelco Co. (Bellefonte, PA, USA). The fibers were conditioned according to Supelco Co. (Bellefonte, PA, USA) instructions. The 2 mL of the samples were divided into separate 5 mL glass vials and hermetically sealed with PTFE/silicone septa. The liquid phase of the sample was kept during equilibration (15 min) and HS-SPME (45 min) in the vials in a water bath at 60 °C below the water surface. A magnetic stirrer was used to perform the extraction with constant stirring of the sample (at 1000 rpm). The SPME fiber was drawn into the needle, removed from the vial, and placed in the injector (250 °C) of a gas chromatograph with a mass spectrometer (GC-MS). After 6 min, the extracted volatiles were thermally desorbed and added directly to the GC column. GC-MS analyses of volatiles were performed using a model 7890A gas chromatograph (Agilent Technologies, Palo Alto, CA, USA) equipped with a HP-5MS capillary column (5% phenylmethylpolysiloxane, Agilent J and W; 30 m × 0.25 mm i.d., coating thickness 0.25 μm) and a model 5977E mass selective detector (MSD) (Agilent Technologies, Palo Alto, CA, USA). The carrier gas was helium (He 1.0 mL min^−1^). The oven temperature was set at 70 °C for 2 min, and then increased to 200 °C (3 °C/min) and held for 15 min. MSD (EI mode) was used at 70 eV with a mass range of 30–300 amu. Retention indices (RIs) were calculated in terms of the retention times of the *n*-alkanes (C9-C25) and their comparison with data from the literature (National Institute of Standards and Technology, Gaithersburg, Maryland, USA) and the mass spectra of the compounds, which matched those of the mass spectral libraries Wiley 9 (Wiley, New York, NY, USA) and NIST 17 (D-Gaithersburg). All extractions (HS-SPME) were performed in duplicate (*n* = 2 replicates), and the results are expressed as mean values of percent composition.

### 3.8. ORAC Assay

The antioxidant capacity of the extracts was determined using the ORAC assay according to the slightly modified procedure described previously [136]. Measurements were performed at λex = 485 nm and λem = 520 nm using the Synergy HTX Multi-Mode microplate reader (BioTek Instruments, Inc., Winooski, VT, USA) converted to 96-well microplates. The 150 μL of fluorescein and 25 μL of the sample (0.075 M phosphate buffer for the blank test and various dilutions of Trolox standard solution) were added to the pores of the microtiter plate. The solutions thus prepared were then thermostated at 37 °C for 30 min. Subsequently, 25 μL of AAPH was added, and the change in fluorescence intensity was measured every minute for 80 min. Extracts were diluted 200-fold, measurements were performed in triplicate, and results were expressed as mean value ± standard error of µM of Trolox equivalents (µM TE).

### 3.9. Statistical Analysis

Statistical analysis was performed using STATISTICA v. 8 software (StatSoft Inc., Tulsa, OK, USA). The experiments were designed as a mixed full factorial design with 3 factors at three levels. The influence of juice (apple, pineapple, orange), extract (*S*, *WT*, *WTS*), and extract concentration (5, 10, and 15%) were considered independent variables. The dependent variables (different sensory characteristics regarding color, odor, flavor, and aroma, and compounds identified by UPLC-MS/MS and ORAC values) were analyzed by analysis of variance (ANOVA) (parametric data) or Kruskal–Wallis test (nonparametric data) after checking the normality of the data set by Shapiro–Wilks test and the homoscedasticity of the residuals by Levene’s test. A statistically significant difference was assumed at a value of *p* ≤ 0.05 (95% confidence interval), and marginal means were compared using Tukey’s HSD test or Kruskal–Wallis test as appropriate. The mean of all results obtained for a given property is listed at the end of the tables as the grand mean.

**Figure A2:**
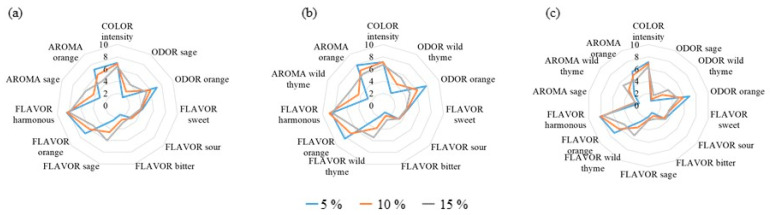
Sensory characteristics of pineapple juice enriched with different concentrations of (**a**) sage, (**b**) wild thyme, and (**c**) wild thyme and sage (3:1, *v*/*v*) mixtures.

**Figure A3:**
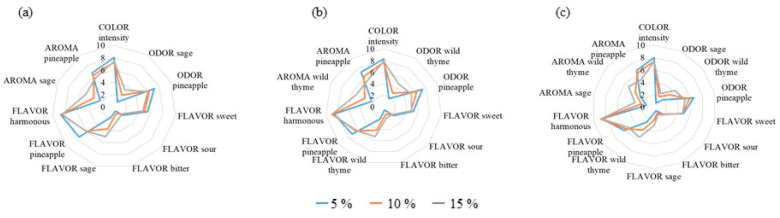
Sensory characteristics of orange juice enriched with different concentrations of (**a**) sage, (**b**) wild thyme, and (**c**) wild thyme and sage (3:1, *v*/*v*) mixtures.

The results of the sensory analysis (Appendix A, Appendix B and Appendix C—Figure A1, Figure A2 and Figure A3) were plotted in polar coordinates, resulting in the representation of the so-called “spider net”, where the intensity of certain features is lowest in the center and increases towards the perimeter of the “net” [137].

## 4. Conclusions

The phenolic content, headspace volatile composition, antioxidant capacity, and sensory analysis of apple, pineapple, and orange juices enriched with wild thyme and sage extracts and their mixture were examined for the first time in the current study. Consumer acceptance of all beverage formulations was quite high, according to the sensory evaluation results. Among the juices, *PJ* had the most intense color and odor. Since the addition of herbal extract had the least impact on sensory properties, the formulations with *PJ* had the most harmonious flavor. *OJ*-based beverages had a stronger sour and bitter flavor, and their *WT* aroma was more dominant. Beverages with *AJ* had the strongest flavor, aroma, and odor of *S* and *WT*. Because the extract odor, flavor, and aroma were used at concentrations as low as 5%, 10%, or 15%, they generally received lower average scores (were less profound) than the juice-derived attributes.

Fruit juices containing herbal extracts had higher phenolic content, which increased antioxidant activity. In addition, the major identified constituents of phenolic compounds were flavonols, mainly rutin, quercetin, and kaempferol derivatives, and hydroxycinnamic acids. The antioxidant activity determined by the ORAC assay was highest in the orange formulations. Monoterpenes, including β-thujone, α-thujone, 1,8-cineole, camphor, carvacrol, 4-terpineol, and thymoquinone, were predominant in both employed fibers of headspace composition, along with other compounds such as sesquiterpenes, aliphatic alcohols, aldehydes, ketones, benzene derivatives, and esters, which have an influence on biological and sensory qualities. The use of wild thyme and sage extracts and their mixture to fortify fruit juices offers enormous potential for the future creation of functional beverages, as each formulation contains a variety of diverse non-volatile and volatile compounds that affect sensory properties.

## Figures and Tables

**Table 1:** Influence of the juice type, extract, and extract concentration on sensory characteristics of functional beverages.

	COLOR	ODOR			FLAVOR								AROMA		
		*S*	*WT*	JUICE	SWEET	SOUR	BITTER	*S*	*WT*	JUICE	HARMONIOUS	OFF	*S*	*WT*	JUICE
Juice	*p <* 0.001 *	*p* = 0.513	*p* = 0.411	*p* = 0.002 *	*p <* 0.001 *	*p <* 0.001 *	*p <* 0.001 *	*p* = 0.513	*p* = 1.000	*p* = 0.411	*p* = 0.070	*p* = 0.125	*p* = 0.800	*p* = 1.000	*p* = 0.411
Apple	6.51 ± 0.24 ^b^	2.08 ± 0.42 ^a^	2.57 ± 0.49 ^a^	4.60 ± 0.21 ^b^	6.56 ± 0.12 ^a^	1.73 ± 0.03 ^b^	0.54 ± 0.07 ^c^	2.38 ± 0.49 ^c^	2.63 ± 0.50 ^a^	6.14 ± 0.20 ^a^	7.95 ± 0.09 ^b^	0.00 ± 0.00 ^a^	2.51 ± 0.54 ^a^	2.65 ± 0.50 ^a,b^	5.98 ± 0.17 ^a^
Pineapple	7.72 ± 0.09 ^a^	1.29 ± 0.28 ^a^	1.81 ± 0.40 ^a^	6.21 ± 0.21 ^a^	4.90 ± 0.11 ^b^	1.94 ± 0.06 ^b^	1.22 ± 0.13 ^b^	1.84 ± 0.41 ^a^	2.65 ± 0.52 ^a^	6.24 ± 0.20 ^a^	8.70 ± 0.18 ^a^	0.00 ± 0.00 ^a^	1.95 ± 0.41 ^a^	2.56 ± 0.49 ^a^	6.06 ± 0.20 ^a^
Orange	6.86 ± 0.09 ^b^	1.35 ± 0.31 ^a^	2.27 ± 0.47 ^a^	6.08 ± 0.23 ^a^	3.90 ± 0.07 ^c^	3.29 ± 0.06 ^a^	1.98 ± 0.14 ^a^	2.19 ± 0.48 ^b^	2.60 ± 0.51 ^a^	6.38 ± 0.25 ^a^	8.25 ± 0.11 ^a,b^	0.02 ± 0.01 ^a^	2.13 ± 0.46 ^a^	2.83 ± 0.53 ^b^	6.11 ± 0.24 ^a^
Extract	*p* = 0.800	*p* < 0.001 *	*p* < 0.001 *	*p* = 0.749	*p* = 0.513	*p* = 0.513	*p* = 1.000	*p* < 0.001 *	*p* < 0.001 *	*p* = 0.135	*p* = 0.411	*p* = 0.125	*p* < 0.001 *	*p* < 0.001 *	*p* = 0.030 *
*S*	7.11 ± 0.17 ^a^	2.97 ± 0.29 ^a^	0.00 ± 0.00 ^b^	5.75 ± 0.26 ^a^	5.29 ± 0.30 ^a^	2.16 ± 0.16 ^a^	1.44 ± 0.21 ^a^	4.19 ± 0.33 ^a^	0.00 ± 0.00 ^b^	6.22 ± 0.21 ^a,b^	8.21 ± 0.17 ^a^	0.02 ± 0.01 ^a^	4.44 ± 0.29 ^a^	0.00 ± 0.00 ^b^	5.94 ± 0.17 ^a,b^
*WT*	6.99 ± 0.23 ^a^	0.00 ± 0.00 ^b^	3.47 ± 0.38 ^a^	5.68 ± 0.34 ^a^	5.10 ± 0.26 ^a^	2.43 ± 0.17 ^b^	1.11 ± 0.18 ^a^	0.00 ± 0.00 ^c^	3.79 ± 0.33 ^a^	6.71 ± 0.23 ^a^	8.46 ± 0.16 ^a^	0.00 ± 0.00 ^a^	0.00 ± 0.00 ^c^	4.08 ± 0.30 ^a^	6.48 ± 0.23 ^a^
*WTS*	6.98 ± 0.18 ^a^	1.75 ± 0.17 ^a^	3.17 ± 0.25 ^a^	5.46 ± 0.21 ^a^	4.98 ± 0.29 ^a^	2.37 ± 0.19 ^b^	1.19 ± 0.15 ^a^	2.22 ± 0.14 ^b^	4.10 ± 0.25 ^a^	5.83 ± 0.16 ^b^	8.24 ± 0.12 ^a^	0.00 ± 0.00 ^a^	2.15 ± 0.13 ^b^	3.95 ± 0.24 ^a^	5.75 ± 0.17 ^b^
Concentration	*p* = 0.451	*p* = 0.055	*p* = 0.015 *	*p* < 0.001 *	*p* = 0.513	*p* = 0.411	*p* = 0.056	*p* = 0.056	*p* = 0.002 *	*p* < 0.001 *	*p* = 0.430	*p* = 0.125	*p* = 0.211	*p* = 0.003 *	*p* < 0.001 *
5	6.89 ± 0.27 ^a^	0.94 ± 0.21 ^a^	1.41 ± 0.26 ^b^	6.60 ± 0.21 ^a^	5.44 ± 0.30 ^a^	2.40 ± 0.17 ^a^	0.78 ± 0.11 ^b^	1.41 ± 0.28 ^a^	1.65 ± 0.30 ^b^	7.14 ± 0.17 ^a^	8.24 ± 0.13 ^a^	0.02 ± 0.01 ^a^	1.60 ± 0.34 ^a^	1.83 ± 0.32 ^b^	6.86 ± 0.15 ^a^
10	6.97 ± 0.13 ^a^	1.62 ± 0.34 ^a^	2.27 ± 0.40 ^a,b^	5.51 ± 0.20 ^b^	5.06 ± 0.28 ^a^	2.29 ± 0.18 ^a^	1.21 ± 0.16 ^a,b^	2.22 ± 0.45 ^b^	2.67 ± 0.46 ^a,b^	6.11 ± 0.13 ^b^	8.46 ± 0.13 ^a^	0.00 ± 0.00 ^a^	2.21 ± 0.46 ^a^	2.67 ± 0.46 ^a,b^	6.03 ± 0.12 ^b^
15	7.22 ± 0.15 ^a^	2.16 ± 0.42 ^a^	2.96 ± 0.59 ^a^	4.78 ± 0.22 ^c^	4.86 ± 0.25 ^a^	2.27 ± 0.18 ^a^	1.76 ± 0.19 ^a^	2.78 ± 0.55 ^c^	3.57 ± 0.62 ^a^	5.51 ± 0.13 ^c^	8.21 ± 0.18 ^a^	0.00 ± 0.00 ^a^	2.78 ± 0.57 ^a^	3.54 ± 0.61 ^a^	5.27 ± 0.14 ^c^
Grand mean	7.03 ± 0.11	1.57 ± 0.20	2.21 ± 0.26	5.63 ± 0.15	5.12 ± 0.16	2.32 ± 0.10	1.25 ± 0.10	2.14 ± 0.26	2.63 ± 0.29	6.25 ± 0.12	8.30 ± 0.09	0.01 ± 0.00	2.20 ± 0.27	2.68 ± 0.29	6.05 ± 0.12

*S* = sage extract; *WT* = wild thyme extract; *WTS* = wild thyme:sage extract (3:1, *v*/*v*). Results are expressed as mean ± SE. * Statistically significant variable at *p* ≤ 0.05. Values with different letters are statistically different at *p* ≤ 0.05.

**Table 2:** Phenolic characterization (g L^−1^ of the sample) of fruit juices and formulations containing sage and wild thyme herbal extracts and their mixtures determined by UPLC/MS-MS analysis.

Compound	RT (Min)	Precursor Ion (*m*/*z*)	Fragment Ions (*m*/*z*)	Tentative Identification		*AJ*	*PJ*	*OJ*	*AS*	*AWT*	*AWTS*	*PS*	*PWT*	*PWTS*	*OS*	*OWT*	*OWTS*
				Hydroxycinnamic acids													
**26**	9.034	359.1	161	Rosmarinic acid **	*p* = 0.045 *	0.27 ± 0.00 ^a^	0.05 ± 0.00 ^a^	0.14 ± 0.00 ^a^	17.40 ± 0.31 ^d^	15.28 ± 0.27 ^c,d^	14.93 ± 0.27 ^c,d^	12.08 ± 0.22 ^b^	16.38 ± 0.29 ^d^	13.93 ± 0.25 ^c,d^	14.93 ± 0.27 ^c,d^	24.52 ± 0.44 ^e^	16.94 ± 0.30 ^d^
**10**	5.173	353	191	Chlorogenic acid **	*p* = 0.013 *	24.08 ± 0.18 ^i^	1.01 ± 0.01 ^a^	2.72 ± 0.02 ^b^	20.14 ± 0.15 ^g^	26.83 ± 0.20 ^j^	23.15 ± 0.17 ^h^	0.85 ± 0.01 ^a^	6.66 ± 0.05 ^d^	5.17 ± 0.04 ^c^	2.76 ± 0.02 ^b^	10.32 ± 0.08 f	7.57 ± 0.06 ^e^
**20**	7.616	193	134	Ferulic acid **	*p* = 0.013 *	0.34 ± 0.00 ^a^	0.51 ± 0.00 ^b^	1.73 ± 0.01 ^g^	1.49 ± 0.01 ^f^	0.89 ± 0.01 ^c^	1.06 ± 0.01 ^d^	1.50 ± 0.01 ^f^	1.31 ± 0.01 ^e^	0.93 ± 0.01 ^c^	2.64 ± 0.02 ^j^	2.12 ± 0.02 ^h^	2.56 ± 0.02 ^i^
**11**	5.575	179	135	Caffeic acid **	*p* = 0.045 *	3.92 ± 0.04 ^c^	1.28 ± 0.01 ^b^	0.46 ± 0.00 ^a^	25.72 ± 0.23 ^j^	11.70 ± 0.11 ^e^	17.17 ± 0.16 ^g^	18.74 ± 0.17 ^h^	9.67 ± 0.11 ^d^	11.62 ± 0.11 ^e^	23.31 ± 0.21 ^i^	11.43 ± 0.10 ^e^	13.24 ± 0.12 ^f^
**15**	7.178	163	119	*p*-Coumaric acid **	*p* = 0.013 *	3.97 ± 0.07 ^f,g^	1.31 ± 0.02 ^b^	0.54 ± 0.01 ^a^	3.83 ± 0.07 ^f,g^	4.35 ± 0.08 ^h^	4.15 ± 0.07 ^g,h^	1.98 ± 0.04 ^c^	2.87 ± 0.05 ^e^	2.52 ± 0.04 ^d^	1.17 ± 0.02 ^b^	1.96 ± 0.03 ^c^	1.75 ± 0.03 ^c^
				Hydroxybenzoic acids													
**29**	11.312	169	125	Gallic acid **	*p* = 0.045 *	0.55 ± 0.01 ^a,b,c^	0.57 ± 0.01 ^b,c^	0.78 ± 0.01 ^e^	0.57 ± 0.01 ^c^	0.56 ± 0.01 ^a,b,c,d^	0.59 ± 0.01 ^d^	0.53 ± 0.01 ^a,b,c^	0.54 ± 0.01 ^a,b,c^	0.54 ± 0.01 ^a,b,c^	0.53 ± 0.01 ^a,b,c^	0.53 ± 0.01 ^a,b,c^	0.54 ± 0.01 ^a,b,c^
**6**	2.783	317	155	3,4-Dihydrobenzoic acid hexoside	*p* = 0.013 *	0.01 ± 0.00 ^a^	0.01 ± 0.00 ^a^	0.01 ± 0.00 ^a^	0.01 ± 0.00 ^a^	0.01 ± 0.00 ^a^	0.01 ± 0.00 ^a^	0.01 ± 0.00 ^a^	0.00 ± 0.00 ^a^	0.01 ± 0.00 ^a^	0.01 ± 0.00 ^a^	0.00 ± 0.00 ^a^	0.00 ± 0.00 ^a^
**9**	4.441	197	182	Syringic acid **	*p* = 0.013 *	0.30 ± 0.00 ^a^	3.04 ± 0.04 ^e^	0.79 ± 0.01 ^b^	1.24 ± 0.02 ^c^	4.15 ± 0.06 ^f,g^	3.93 ± 0.05 ^f^	4.23 ± 0.06 ^f,g^	8.55 ± 0.12 ^i^	8.20 ± 0.11 ^h^	1.33 ± 0.02 ^c^	4.39 ± 0.06 ^g^	2.53 ± 0.03 ^d^
**7**	3.597	153	109	Protocatechuic acid **	*p* = 0.013 *	0.36 ± 0.01 ^c^	0.16 ± 0.00 ^a^	0.28 ± 0.01 ^b^	0.6 ± 0.01 ^e^	0.95 ± 0.02 ^h,i^	0.71 ± 0.01 ^f^	0.57 ± 0.01 ^e^	1.21 ± 0.02 ^j^	0.82 ± 0.02 ^g,h^	0.45 ± 0.01 ^d^	0.89 ± 0.02 ^g,h,i^	0.58 ± 0.01 ^e^
**8**	4.417	137	93	*p*-Hydroxybenzoic acid	*p* = 0.013 *	0.43 ± 0.01 ^a^	0.71 ± 0.01 ^b^	0.46 ± 0.01 ^a^	1.12 ± 0.02 ^c^	2.82 ± 0.04 ^h^	2.15 ± 0.03 ^g^	1.24 ± 0.02 ^d^	2.72 ± 0.04 ^h^	1.80 ± 0.02 ^f^	1.26 ± 0.02 ^d^	2.14 ± 0.03 ^g^	1.54 ± 0.02 ^e^
				Flavones													
**12**	6.287	449	329	Luteolin-6-C-hexoside	*p* = 0.013 *	0.00 ± 0.00 ^a,b^	0.01 ± 0.00 ^c^	0.02 ± 0.00 ^e^	0.00 ± 0.00 ^a,b^	0.01 ± 0.00 ^d^	0.01 ± 0.00 ^c^	0.00 ± 0.00 ^b,c^	0.01 ± 0.00 ^d^	0.01 ± 0.00 ^c^	0.02 ± 0.00 ^f^	0.03 ± 0.00 ^g^	0.02 ± 0.00 ^g^
**18**	7.24	287	153	Luteolin **	*p* = 0.013 *	0.16 ± 0.00 ^a^	0.21 ± 0.01 ^a^	0.52 ± 0.01 ^b^	2.44 ± 0.06 ^g^	1.95 ± 0.05 ^f^	1.45 ± 0.04 ^d^	1.86 ± 0.05 ^f^	1.14 ± 0.03 ^c^	1.50 ± 0.04 ^d,e^	1.38 ± 0.03 ^d,e^	1.44 ± 0.03 ^d,e^	1.40 ± 0.03 ^d,e^
**28**	11.285	271	153	Apigenin **	*p* = 0.045 *	0.06 ± 0.00 ^b^	0.02± 0.00 ^a^	0.03 ± 0.00 ^a^	0.09 ± 0.00 ^b^	0.27 ± 0.01 ^e^	0.07 ± 0.00 ^b^	0.11 ± 0.00 ^c^	0.15 ± 0.00 ^d^	0.07 ± 0.00 ^b^	0.07 ± 0.00 ^b^	0.13 ± 0.00 ^c^	0.07 ± 0.00 ^b^
**1**	0.805	579	459	Apigenin-6-C-(*O*-deoxyhexosyl)-hexoside	*p* = 0.045 *	0.00 ± 0.00 ^c^	0.00 ± 0.00 ^c^	0.0 ± 0.00 ^d^	0.0 ± 0.00 ^d^	0.00 ± 0.00 ^a^	0.00 ± 0.00 ^a^	0.0 ± 0.00 ^e^	0.01 ± 0.00 ^e^	0.01 ± 0.00 ^g^	0.01 ± 0.00 ^f^	0.01 ± 0.00 ^f^	0.00 ± 0.00 ^b^
				Flavonols													
**21**	8.082	611	303	Rutin **	*p* = 0.045 *	1.4 ± 0.03 ^a^	0.19 ± 0.00 ^a^	101.17 ± 0.25 ^f^	1.49 ± 0.00 ^a^	5.25 ± 0.11 ^a,b,c^	5.26 ± 0.01 ^a,b,c^	1.01 ± 0.00 ^a^	7.77 ± 0.16 ^a,b,c^	5.20 ± 0.01 ^a,b,c^	93.26 ± 1.90 ^e^	81.80 ± 0.21 ^d^	107.58 ± 2.20 ^g^
**14**	7.075	465	303.1	Quercetin-3-glucoside **	*p* = 0.045 *	3.71 ± 0.07 ^b^	0.34 ± 0.01 ^a^	4.54 ± 0.09 ^c^	4.74 ± 0.09 ^c,d^	5.54 ± 0.11 ^d^	5.41 ± 0.11 ^d^	3.70 ± 0.07 ^b^	7.06 ± 0.14 ^e^	5.68 ± 0.11 ^d^	5.21 ± 0.10 ^d^	9.82 ± 0.19 ^f^	7.05 ± 0.14 ^e^
**25**	8.986	449	303	Quercetin-3-rhamnoside	*p* = 0.013 *	2.96 ± 0.04 ^c^	0.09 ± 0.00 ^a^	1.77 ± 0.03 ^b^	3.31 ± 0.05 ^d^	7.94 ± 0.12 ^h^	4.97 ± 0.07 ^f^	0.19 ± 0.00 ^a^	4.46 ± 0.07 ^e^	2.65 ± 0.04 ^c^	1.76 ± 0.03 ^b^	5.97 ± 0.09 ^g^	4.28 ± 0.06 ^e^
**23**	8.49	435	303	Quercetin-3-pentoside	*p* = 0.013 *	3.37 ± 0.07 ^f^	0.04 ± 0.00 ^a^	0.06 ± 0.00 ^a^	3.01 ± 0.06 ^d,e^	3.10 ± 0.06 ^d,e^	2.83 ± 0.06 ^d^	0.06 ± 0.00 ^a^	0.51 ± 0.01 ^b,c^	0.29 ± 0.01 ^b^	0.05 ± 0.00 ^a^	0.58 ± 0.01 c	0.40 ± 0.01 ^b,c^
**16**	7.178	595	287	Kaempferol-3-rutinoside	*p* = 0.045 *	0.10 ± 0.00 ^a^	0.29 ± 0.00 ^a^	3.20 ± 0.0 ^b^	11.26 ± 0.02 ^j^	4.10 ± 0.06 ^d^	5.50 ± 0.01 ^f^	9.80 ± 0.01 ^i^	3.76 ± 0.05 ^c^	5.30 ± 0.01 ^f^	9.40 ± 0.14 ^h^	4.69 ± 0.01 ^e^	6.22 ± 0.09 ^g^
**17**	7.208	449	287	Kaempferol-3-*O*-hexoside	*p* = 0.013 *	0.70 ± 0.02 ^a^	0.22 ± 0.00 ^a^	9.70 ± 0.15 ^b,c^	34.24 ± 0.52 ^h^	8.31 ± 0.13 ^b,c^	14.78 ± 0.23 ^e^	25.92 ± 0.39 ^g^	8.13 ± 0.12 ^b^	12.19 ± 0.19 ^d^	26.98 ± 0.41 ^g^	17.99 ± 0.27 ^f^	12.01 ± 0.18 ^d^
**4**	1.364	433	287	Kaempferol-3-*O*-deoxyhexoside	*p* = 0.013 *	0.01 ± 0.00 ^c,d,e^	0.02 ± 0.00 ^h^	0.02 ± 0.00 ^f^	0.00 ± 0.00 ^a,b^	0.00 ± 0.00 ^a,b,c^	0.01 ± 0.00 ^d,e^	0.02 ± 0.00 ^g^	0.03 ± 0.00 ^i^	0.03 ± 0.00 ^h^	0.01 ± 0.00 ^b,c^	0.01 ± 0.00 ^b,c,d^	0.01 ± 0.00 ^b,c,d^
**22**	8.355	419	287	Kaempferol-3-*O*-pentoside	*p* = 0.013 *	0.17 ± 0.00 ^c,d^	0.07 ± 0.00 ^a^	0.13 ± 0.00 ^b,c,d^	0.16 ± 0.00 ^c,d^	0.56 ± 0.01 ^g^	0.33 ± 0.00 ^f^	0.12 ± 0.00 ^b,c^	0.26 ± 0.01 ^e^	0.19 ± 0.00 ^d^	0.14 ± 0.00 ^b,c,d^	0.32 ± 0.01 ^f^	0.25 ± 0.01 ^e^
**13**	6.785	319	273	Myricetin **	*p* = 0.013 *	0.44 ± 0.00 ^b^	0.22 ± 0.00 ^a^	1.69 ± 0.01 ^h^	1.99 ± 0.01 ^i^	0.72 ± 0.01 ^c^	1.01 ± 0.01 ^e^	1.28 ± 0.01 ^g^	1.17 ± 0.01 ^f^	0.80 ± 0.00 ^d^	1.69 ± 0.01 ^h^	2.04 ± 0.01 ^j^	2.40 ± 0.01 ^k^
**19**	7.575	479	317	Isorhamnetin-3-hexoside	*p* = 0.013 *	1.02 ± 0.03 ^b,c^	1.12 ± 0.03 ^b,c^	0.50 ± 0.01 ^a^	1.75 ± 0.04 ^e^	1.58 ± 0.04 ^d,e^	0.93 ± 0.02 ^b^	1.63 ± 0.04 ^d,e^	1.50 ± 0.04 ^d,e^	1.39 ± 0.04 ^d^	1.54 ± 0.04 ^d,e^	1.04 ± 0.04 ^b,c^	0.87 ± 0.03 ^b^
				Flavan-3-ols													
**3**	1.244	291	139	Catechin **	*p* = 0.045 *	0.38 ± 0.00 ^c^	0.23 ± 0.00 ^a^	0.49 ± 0.00 ^e^	0.48 ± 0.00 ^e^	0.62 ± 0.01 ^f^	0.63 ± 0.01 ^f^	0.44 ± 0.00 ^d^	0.25 ± 0.00 ^a^	0.32 ± 0.00 ^b^	0.61 ± 0.01 ^f^	0.47 ± 0.00 ^e^	0.48 ± 0.00 ^e^
**5**	1.801	291	123	Epicatechin	*p* = 0.013 *	0.90 ± 0.01 ^h^	0.16 ± 0.00 ^a^	0.50 ± 0.01 ^d,e^	0.30 ± 0.00 ^b^	0.52 ± 0.01 ^e^	0.47 ± 0.01 ^d^	0.26 ± 0.00 ^b^	0.40 ± 0.01 ^c^	0.24 ± 0.00 ^b^	0.57 ± 0.01 ^f^	0.68 ± 0.01 ^g^	0.38 ± 0.01 ^c^
**27**	9.884	459	139	Epigalocatechin gallate **	*p* = 0.013 *	0.02 ± 0.00 ^a,b,c^	0.02 ± 0.00 ^a,b^	0.29 ± 0.00 ^g^	0.03 ± 0.00 ^b,c,d^	0.03 ± 0.00 ^b,c,d^	0.03 ± 0.00 ^a,b,c^	0.01 ± 0.00 ^a^	0.24 ± 0.00 ^e^	0.04 ± 0.00 ^c,d^	0.32 ± 0.00 ^h^	0.27 ± 0.00 ^f^	0.34 ± 0.00 ^i^
**24**	8.78	442.9	139	Epicatechin gallate **	*p* = 0.013 *	0.10 ± 0.00 ^b,c^	0.14 ± 0.00 ^f^	0.07 ± 0.00 ^a^	0.14 ± 0.00 ^f^	0.16 ± 0.00 ^g^	0.12 ± 0.00 ^d^	0.13 ± 0.00 ^e^	0.18 ± 0.00 ^h^	0.16 ± 0.00 ^g^	0.14 ± 0.00 ^f^	0.10 ± 0.00 ^c^	0.09 ± 0.00 ^b^
				Proanthocyanidins													
**2**	1.172	865	575	Procyanidin trimer **	*p* < 0.013 *	1.57 ± 0.02 ^j^	1.59 ± 0.02 ^j^	0.40 ± 0.01 ^e,f^	0.18 ± 0.00 ^a,b,c^	0.14 ± 0.00 ^a,b,c^	1.31 ± 0.02 ^i^	0.51 ± 0.01 ^g^	0.21 ± 0.00 ^a,b,c,d^	1.18 ± 0.02 ^h^	0.28 ± 0.00 ^c,d,e^	0.23 ± 0.00 ^b,c,d^	0.33 ± 0.01 ^d,e,f^

*AJ* = apple juice; *PJ* = pineapple juice; *OJ* = orange juice; *AS* = apple juice + sage extract; *AWT* = apple juice + wild thyme extract; *AWTS* = apple juice + wild thyme:sage extract (3:1, *v*/*v*); *PS* = pineapple juice + sage extract; *PWT* = pineapple juice + wild thyme extract; *PWTS* = pineapple juice + wild thyme:sage extract (3:1, *v*/*v*); *OS* = orange juice + sage extract; *OWT* = orange juice + wild thyme extract; *OWTS* = orange juice + wild thyme:sage extract (3:1, *v*/*v*). The results are expressed as mg L^−1^ as mean ± SE. ** Identification confirmed using authentic standards. * Statistically significant variable at *p* ≤ 0.05. The values with different letters within a row are statistically different at *p* ≤ 0.05.

**Table 3:** The headspace chemical composition (%) of fruit juices and formulations containing sage and wild thyme herbal extracts and their mixture as determined by HS-SPME/GC-MS analysis on polydimethylsiloxane/divinylbenzene (PDMS/DVB) fiber.

No.	Compound	RI	*AJ*	*PJ*	*OJ*	*AS*	*AWT*	*AWTS*	*PS*	*PWT*	*PWTS*	*OS*	*OWT*	*OWTS*
Aliphatic alcohols														
1.	Ethanol	˂900	61.73	-	0.14	0.88	2.99	2.12	1.64	4.18	-	-	0.84	0.47
2.	3-Methylbutan-1-ol	˂900	4.62	-	-	-	0.53	0.08	0.13	0.25	0.76	-	-	-
3.	2-Methylpropan-1-ol	˂900	6.53	-	-	-	-	-	-	-	-	-	-	-
4.	Butan-2,3-diol	˂900	-	15.25	-	0.41	0.96	0.26	0.17	0.25	0.40	0.05	0.03	-
5.	2-Furanmethanol	˂900	3.13	-	-	-	-	-	-	-	-	-	-	-
6.	(*Z*)-Hex-3-en-1-ol	˂900	-	-	-	-	0.32	0.13	-	0.32	-	-	-	-
7.	Oct-1-en-3-ol	984	-	-	-	0.29	7.19	2.95	0.23	3.12	2.57	0.05	0.25	0.17
8.	Octan-3-ol	998	-	-	-	0.26	1.32	-	-	0.46	-	-	-	-
9.	Octan-1-ol	1076	-	-	0.12	-	-	-	-	-	-	0.14	0.13	0.11
Aldehydes														
10.	Furfural	˂900	0.95	1.71	-	-	-	-	-	-	-	-	-	-
11.	Octanal	1007	-	-	0.05	-	-	-	-	-	-	0.05	0.07	0.05
12.	Decanal	1210	-	-	0.25	-	-	-	-	-	-	0.27	0.31	0.30
Alkanes														
13.	Pentadecane	1500	-	-	-	0.32	0.26	-	-	-	-	-	-	-
Esters														
14.	γ-Butyrolactone	923	-	1,16	-	-	-	-	-	-	-	-	-	-
15.	γ-Caprolactone	1062	-	9.06	-	-	-	-	-	-	-	-	-	-
Ketones														
16.	Propan-2-one	˂900	13.64	61.82	-	-	-	-	-	-	-	-	-	-
17.	Acetoin	˂900	-	-	-	-	1.10	0.09	0.41	0.65	1.82	-	-	-
18.	Octan-3-one	991	-	-	-	-	0.96	0.39	-	0.40	-	-	0.03	-
Monoterpenes														
19.	α-Thujene	937	-	-	-	-	-	-	-	0.18	-	-	-	-
20.	α-Pinene	945	-	-	0.20	-	-	-	-	-	-	0.17	0.20	0.20
21.	Sabinene	982	-	-	-	-	-	-	-	1.82	-	-	-	-
22.	β-Myrcene	996	-	-	1.76	-	-	-	-	0.44	-	1.27	1.53	1.49
23.	α-Phellandrene	1011	-	-	0.08	-	0.21	0.11	-	0.14	-	0.07	0.10	0.07
24.	δ-Car-3-ene	1017	-	-	0.15	-	-	-	-	-	-	0.12	0.15	0.14
25.	α-Terpinene	1023	-	-	0.09	-	0.31	0.18	-	0.32	-	0.07	0.09	0.08
26.	*p*-Cymene	1032	-	-	-	0.93	0.71	0.97	2.17	1.82	0.63	0.12	0.05	0.08
27.	Limonene	1037	-	-	92.24	-	0.49	0.09	0.28	-	-	77.19	85.45	84.41
28.	β-Phellandrene	1037	-	-	-	-	-	0.06	-	0.37	-	-	-	-
29.	1,8-Cineole	1039	-	-	-	13.28	1.48	9.03	11.35	0.69	8.02	2.28	-	0.95
30.	(*Z*)-β-Ocymene	1044	-	-	-	-	-	-	0.91	12.18	-	-	-	-
31.	(*E*)-β-Ocymene	1055	-	-	0.06	-	-	0.20	3.43	0.39	-	0.04	0.05	0.05
32.	γ-Terpinene	1066	-	-	0.18	0.14	0.62	0.31	0.37	2.29	-	0.14	0.17	0.17
33.	α-Terpinolene	1093	-	-	1.72	-	-	-	4.32	-	-	0.10	0.12	0.13
34.	Linalool	1103	-	-	-	1.06	7.92	3.71	0.96	6.83	3.15	2.04	2.17	1.93
35.	β-Thujone	1112	-	-	-	34.72	1.91	21.93	30.63	0.97	17.74	4.78	0.08	1.54
36.	α-Thujone	1123	-	-	-	18.19	0.91	10.79	15.51	0.47	8.31	2.19	-	0.69
37.	*trans*-Sabinol	1147	-	-	-	0.25	-	-	-	0.11	-	-	-	-
38.	Camphor	1152	-	-	-	16.38	5.72	13.10	14.77	2.83	12.97	3.17	0.28	1.12
39.	Borneol	1172	-	-	-	3.28	10.19	5.97	3.02	5.30	5.94	0.61	0.52	0.47
40.	4-Terpineol	1182	-	-	0.68	1.92	15.28	7.46	1.74	8.74	7.14	1.16	1.62	1.34
41.	α-Terpineol	1195	-	-	0.46	0.32	1.64	0.91	0.27	0.91	0.94	0.65	0.65	0.55
42.	*cis*-Dihydrocarvone	1200	-	-	-	-	0.13	0.06	-	0.14	-	-	0.05	-
43.	*trans*-Dihydrocarvone	1209	-	-	-	-	0.24	-	-	0.15	-	-	-	-
44.	Carvone	1250	-	-	0.06	-	-	-	-	-	-	0.07	-	0.07
45.	Geraniol	1260	-	-	-	-	7.63	3.06	-	6.40	2.63	-	0.56	
46.	Thymol	1297	-	-	-	-	6.56	2.54	-	5.31	3.01	-	0.46	0.27
47.	Carvacrol	1307	-	-	-	-	11.74	4.91	-	9.09	5.43	-	0.90	0.51
48.	Eugenol	1363	-	-	-	-	0.48	0.10	-	0.33	-	-	0.03	-
49.	Nerol	1234	-	-	-	-	0.68	0.27	-	0.40	-	-	0.08	0.08
50.	*p*-Cymene-2,5-dione (Thymoquinone)	1256	-	-	-	-	-	-	-	8.94	-	-	-	-
51.	Carvacryl methyl ether	1236	-	-	-	-	-	-	-	0.83	-	-	-	-
52.	Methyl thymyl ether	1241	-	-	-	-	-	-	-	1.54	-	-	-	-
Benzene derivatives														
53.	4-Vinylphenol	1225	-	-	-	-	0.72	0.34	-	0.33	0.51	-	-	-
54.	2-Phenylethanol	1118	0.40	-	-	-	0.31	-	-	-	-	-	-	-
55.	Thujol	1141	-	-	-	0.45	-	-	-	-	-	-	-	-
56.	*p*-Cymen-8-ol	1190	-	-	-	0.15	0.69	0.44	0.35	0.37	0.44	-	-	-
Sesquiterpenes														
57.	*trans*-β-Caryophyllene	1423	-	-	-	-	-	-	-	0.77	-	-	0.06	-
58.	Eremophilene	1496	-	-	1.00	-	-	-	-	-	-	0.94	0.96	0.94
59.	δ-Cadinene	1528	-	-	0.11	-	-	-	-	-	-	0.10	0.11	0.10

*AJ* = apple juice; *PJ* = pineapple juice; *OJ* = orange juice; *AS* = apple juice + sage extract; *AWT* = apple juice + wild thyme extract; *AWTS* = apple juice + wild thyme:sage extract (3:1, *v*/*v*); *PS* = pineapple juice + sage extract; *PWT* = pineapple juice + wild thyme extract; *PWTS* = pineapple juice + wild thyme:sage extract (3:1, *v*/*v*); *OS* = orange juice + sage extract; *OWT* = orange juice + wild thyme extract; *OWTS* = orange juice + wild thyme:sage extract (3:1, *v*/*v*). The results are expressed as the mean value of percentage composition. Standard deviation was lower than 10%.

**Table 4:** The headspace chemical composition (%) of fruit juices and formulations containing sage and wild thyme herbal extracts and their mixture as determined by HS-SPME/GC-MS analysis on divinylbenzene/carboxen/polydimethylsiloxane (DVB/CAR/PDMS) fiber.

No.	Compond	RI	*AJ*	*PJ*	*OJ*	*AS*	*AWT*	*AWTS*	*PS*	*PWT*	*PWTS*	*OS*	*OWT*	*OWTS*
Aliphatic alcohols														
1.	Ethanol	˂900	47.95	-	-	1.97	5.40	-	5.46	2.73	15.36	0.46	0.97	0.46
2.	3-Methylbutan-1-ol	˂900	3.90	-	-	-	1.07	-	0.08	0.10	1.74	-	-	-
3.	2-Methylpropan-1-ol	˂900	18.53	-	-	-	-	-	-	-	-	-	-	-
4.	Butan-2,3-diol	˂900	-	4.76	-	0.23	0.32	0.30	-	-	-	-	-	-
5.	2-Furanmethanol	˂900	2.06	-	-	-	-	-	-	-	-	-	-	-
6.	(*Z*)-Hex-3-en-1-ol	˂900	-	-	-	-	0.49	0.25	-	-	-	-	-	-
7.	Oct-1-en-3-ol	984	-	-	-	0.31	5.54	3.18	-	3.15	2.14	-	0.28	0.16
8.	Octan-3-ol	998	-	-	-	0.12	1.03	-	-	-	-	-	-	-
Aldehydes														
9.	Furfural	˂900	2.91	3.63	-	-	-	-	-	-	-	-	-	-
10.	Octanal	1007	-	-	0.11	-	-	-	-	-	-	0.12	-	-
11.	Decanal	1210	-	-	0.40	-	-	-	-	-	-	0.51	0.53	0.42
12.	3-Methylbutanal	˂900	-	80.11	-	-	-	-	-	-	-	-	-	-
	Ketones													
13.	Propan-2-one	˂900	14.04	-	-	-	-	-	-	-	-	-	-	-
14.	Acetoin	˂900	-	-	-	-	1.85	-	0.34	0.64	3.23	-	-	-
15.	Octan-3-one	991	-	-	-	-	0.60	-	-	0.45	-	-	-	-
Monoterpenes														
16.	α-Pinene	945	-	-	0.23	-	-	-		-	-	0.13	0.16	0.18
17.	β-Myrcene	996	-	-	1.31	-	-	-	-	0.32	-	0.81	0.98	1.04
18.	α-Phellandrene	1011	-	-	0.09	-	-	-	-	-	-	0.07	0.11	0.09
19.	δ-Car-3-ene	1017	-	-	0.13	-	-	-	-	-	-	0.09	0.11	0.11
20.	α-Terpinene	1023	-	-	0.07	-	-	-	-	-	-	-	0.09	0.06
21.	*p*-Cymene	1032	-	-	-	1.24	-	0.72	1.30	0.45	-	0.18	0.06	0.11
22.	Limonene	1037	-	-	91.12	-	-	-	-	-	-	72.50	81.43	82.30
23.	1,8-Cineole	1039	-	-	-	13.06	0.50	7.98	12.16	-	4.86	3.01	-	1.72
24.	(*Z*)-β-Ocymene	1044	-	-	-	-	-	-	-	1.43	-	-	-	-
25.	(*E*)-β-Ocymene	1055	-	-	0.04	-	-	-	-	-	-	-	0.07	-
26.	γ-Terpinene	1066	-	-	0.13	-	-	-	0.35	0.71	-	0.11	0.12	0.16
27.	α-Terpinolene	1093	-	-	0.13	-	-	-	1.52	5.46	-	0.08	0.18	0.15
28.	Linalool	1103	-	-	1.46	1.13	7.15	4.40	1.12	-	3.06	2.45	2.70	1.79
29.	β-Thujone	1112	-	-	-	35.68	1.16	20.11	31.65	0.64	10.83	5.63	-	1.50
30.	α-Thujone	1123	-	-	-	18.06	-	9.30	15.17	-	4.39	2.52	0.12	0.68
31.	Camphor	1152	-	-	-	17.38	3.96	13.42	16.95	2.60	10.05	3.67	0.34	1.12
32.	Borneol	1172	-	-	-	3.05	7.09	5.96	3.54	5.32	5.57	0.68	0.48	0.39
33.	4-Terpineol	1182	-	-	0.71	2.67	15.89	9.78	2.55	10.95	7.94	1.58	2.23	1.37
34.	α-Terpineol	1195	-	-	0.65	0.50	2.21	1.43	0.48	1.45	1.25	1.05	1.10	0.74
35.	Carvone	1250	-	-	-	-	-	-	-	-	-	0.11	-	0.04
36.	Geraniol	1260	-	-	-	-	9.24	-	-	9.81	4.22	-	0.82	0.29
37.	Thymol	1297	-	-	-	-	9.03	4.27	-	9.80	3.88	-	0.67	0.28
38.	Carvacrol	1307	-	-	-	-	17.83	9.67	-	18.97	9.14	-	1.48	0.67
39.	Eugenol	1363	-	-	-	-	0.83	0.30	-	0.67	-	-	-	-
40.	Nerol	1234	-	-	-	-	-	-	-	-	-	-	0.11	-
41.	*p*-Cymene-2,5-dione (Thymoquinone)	1256	-	-	-	-	-	-	-	13.78	5.45	-	0.20	-
42.	Methyl thymyl ether	1241	-	-	-	-	-	-	-	0.84	-	-	-	-
Benzene derivatives														
43.	4-Vinylphenol	1225	-	-	-	-	0.17	-	-	-	-	-	-	-
44.	Benzenmethanol	1041	-	-	-	-	0.33	-	-	-	-	-	-	-
45.	Thujol	1141	-	-	-	0.45	-	-	-	-	-	-	-	-
46.	*p*-Cymen-8-ol	1190	-	-	-	-	0.81	0.25	-	0.63	-	-	0.10	-
Sesquiterpenes														
47.	Ledene	1498	-	-	1.08	-	-	-	-	-	-	-	-	-
48.	Eremophilene	1496	-	-	-	-	-	-	-	-	-	1.42	1.48	1.25
49.	Δ-cadinene		-	-	0.12	-	-	-	-	-	-	0.17	0.17	0.16

*AJ* = apple juice; *PJ* = pineapple juice; *OJ* = orange juice; *AS* = apple juice + sage extract; *AWT* = apple juice + wild thyme extract; *AWTS* = apple juice + wild thyme:sage extract (3:1, *v*/*v*); *PS* = pineapple juice + sage extract; *PWT* = pineapple juice + wild thyme extract; *PWTS* = pineapple juice + wild thyme:sage extract (3:1, *v*/*v*); *OS* = orange juice + sage extract; *OWT* = orange juice + wild thyme extract; *OWTS* = orange juice + wild thyme:sage extract (3:1, *v*/*v*). The results are expressed as the mean value of percentage composition. Standard deviation was lower than 10%.

**Table 5:** Antioxidant capacity of fruit juices and formulations containing sage and wild thyme herbal extracts and their mixtures determined by ORAC assay.

	*AJ*	*PJ*	*OJ*	*AS*	*AWT*	*AWTS*	*PS*	*PWT*	*PWTS*	*OS*	*OWT*	*OWTS*
**ORAC** (µM TE) * p = 0.001	3120.81 ± 162.79 ^b^	3082.01 ± 449.90 ^b^	8060.73 ± 283.92 ^a,b^	9981.77 ± 644.10 ^a,b^	7818.99 ± 44.93 ^a,b^	7397.93 ± 85.56 ^a,b^	12,633.20 ± 193.52 ^a^	7765.70 ± 369.78 ^a,b^	8951.84 ± 261.46 ^a,b^	22,925.39 ± 358.43 ^a^	9623.34 ± 184.14 ^a,b^	5670.34 ± 65.88 ^a,b^

*AJ* = apple juice; *PJ* = pineapple juice; *OJ* = orange juice; *AS* = apple juice + sage extract; *AWT* = apple juice + wild thyme extract; *AWTS* = apple juice + wild thyme:sage extract (3:1, *v*/*v*); *PS* = pineapple juice + sage extract; *PWT* = pineapple juice + wild thyme extract; *PWTS* = pineapple juice + wild thyme:sage extract (3:1, *v*/*v*); *OS* = orange juice + sage extract; *OWT* = orange juice + wild thyme extract; *OWTS* = orange juice + wild thyme:sage extract (3:1, *v*/*v*). The results are expressed as µM TE as mean ± SE. * Statistically significant variable at *p* ≤ 0.05. The values with different letters are statistically different at *p* ≤ 0.05.

**Table 6:** Beverage formulations composed of fruit juices and enriched with herbal extracts.

Juice	Extract	Label
Apple	sage	*AS*
	wild thyme	*AWT*
	wild thyme:sage (3:1, *v*/*v*)	*AWTS*
Pineapple	sage	*PS*
	wild thyme	*PWT*
	wild thyme:sage (3:1, *v*/*v*)	*PWTS*
Orange	sage	*OS*
	wild thyme	*OWT*
	wild thyme:sage (3:1, *v*/*v*)	*OWTS*

**Figure A1:**
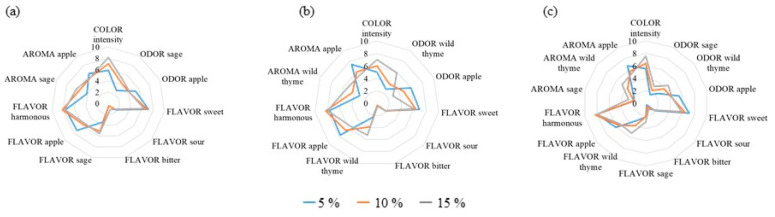
Sensory characteristics of apple juice enriched with different concentrations of (**a**) sage, (**b**) wild thyme, and (**c**) wild thyme and sage (3:1, *v*/*v*) mixtures.

## Data Availability

The data presented in this study are available in this manuscript.
